# 准确度曲线评估在尿酚定量检测方法建立中的应用

**DOI:** 10.3724/SP.J.1123.2025.04031

**Published:** 2026-03-08

**Authors:** Xiaoning LIU, Yuanheng SHEN, Weifeng TANG, Junjie AO, Junxia LIU, Li ZHAO, Lili YUAN, Xian HUANG, Qianlong ZHANG

**Affiliations:** 1.上海交通大学医学院附属新华医院，环境与儿童健康教育部和上海市重点实验室，上海 200092; 1. Xinhua Hospital Affiliated to Shanghai Jiao Tong University School of Medicine，Ministry of Education-Shanghai Key Laboratory of Children’s Environmental Health，Shanghai 200092，China; 2.上海交通大学医学院附属新华医院检验科，上海 200092; 2. Department of Laboratory Medicine，Xinhua Hospital Affiliated to Shanghai Jiao Tong University School of Medicine，Shanghai 200092，China

**Keywords:** 准确度评估, *β*-容许区间, 尿酚, 高效液相色谱-串联质谱, accuracy assessment, *β*-expectation tolerance intervals, urinary phenols, high performance liquid chromatography-tandem mass spectrometry （HPLC-MS/MS）

## Abstract

准确可靠的检测方法是确保结果可信的关键。受法规监管的检测实验室通常采用标准方法，仅需满足标准特征参数要求即可提供服务。然而，科研实验室多使用自建非标方法，因缺乏统一确认标准，方法准确度评估尤为重要。传统评估多依赖单项特性参数，存在标准不一、结果解释复杂等局限。近年来，法国药学科学与技术学会委员会（Société Française des Sciences et Techniques Pharmaceutiques， SFSTP）提出了一种统一、综合且易判别的准确度曲线评估方法。本研究以20种尿酚的高效液相色谱-串联质谱（HPLC-MS/MS）检测方法为例，详细介绍该方法，并与传统方法进行对比，探讨其推广应用的可行性。本研究采用SFSTP方法逐步评估检测方法的正确度和精密度，计算*β*-容许区间，绘制准确度曲线，以综合评价方法的准确性和可靠性，并与传统评估方法进行对比。研究结果表明，在低、中、高3个浓度水平下，20种尿酚检测方法的批内和批间相对标准偏差（RSD）分别为2.8%~10.7%和3.3%~14.4%，合成后的中间精密度RSD为4.9%~16.6%；检测结果的相对误差为-25.3%~13.0%，按传统单项特征参数评估，20种尿酚的准确度均符合要求。而准确度曲线显示，在95%的置信水平下，13种尿酚（如双酚AF、双酚B等）在不同浓度水平的*β*-容许区间全部落在可接受限［-30%，30%］内，而7种尿酚（如对羟基苯甲酸苄酯、二苯酮-8等）在低或中浓度水平的*β*-容许区间超出了可接受范围。综上，准确度曲线评估表明，所建立的20种尿酚的HPLC-MS/MS检测方法具有较高的准确性和可靠性。与传统单项参数评估相比，该方法无需增加实验成本，仅通过一次统计分析即可清晰判断方法性能，兼具综合性（整合方法特性与风险评估）、结果直观易解释、标准统一等优势，适合广泛推广应用。

## Abstract (en)

Accurate and reliable analytical methods are fundamental to ensuring the validity and reproducibility of scientific results， particularly in research involving trace-level contaminants in biological samples. While standardized methods regulated by official guidelines are commonly used in certified laboratories， research laboratories often rely on in-house or self-developed non-standard methods tailored for specific studies. These methods， however， frequently lack harmonized validation criteria， especially regarding accuracy evaluation. Traditionally， method accuracy is assessed through single performance indicators such as recovery， relative standard deviation （RSD）， or bias. These indicators， although useful， may vary widely in their interpretation and offer limited insight into overall method performance， particularly when comparing between laboratories or over different concentration ranges. To address these limitations， the Société Française des Sciences et Techniques Pharmaceutiques （SFSTP） introduced the accuracy profile approach. This method integrates both trueness and precision using a statistical model to generate *β*-expectation tolerance intervals， allowing a visual and comprehensive representation of whether an analytical method meets predefined acceptance criteria across multiple concentration levels. Unlike traditional evaluations， which may obscure marginal performance failures， the accuracy profile provides a single， unified framework that simplifies interpretation and decision-making. In this study， we applied the SFSTP accuracy profile approach to validate a high performance liquid chromatography-tandem mass spectrometry （HPLC-MS/MS） method for quantifying 20 urinary phenols. Method performance was assessed at three concentration levels （low， medium， and high） and the results were compared with conventional validation methods. The intra-batch RSD values ranged from 2.8% to 10.7%， and inter-batch RSD values ranged from 3.3% to 14.4%. The pooled intermediate precision RSD ranged from 4.9% to 16.6%， indicating acceptable reproducibility. Relative errors were between -25.3% and 13.0%， According to traditional evaluation using single parameters， the accuracy of all 20 phenol measurements met standard criteria. However， accuracy profile analysis revealed that only 13 phenols， such as bisphenol AF and bisphenol B， had *β*-expectation tolerance intervals entirely within the ±30% acceptance limits across all tested concentration levels. The remaining seven phenols， including benzylparaben and benzophenone-8， exceeded acceptable limits at low or medium concentrations， suggesting areas for method optimization. In conclusion， this study demonstrates that the HPLC-MS/MS method provides reliable quantitative results for the majority of tested urinary phenols. More importantly， the SFSTP accuracy profile offers a superior alternative to conventional validation methods by combining accuracy parameters and risk analysis into a coherent， statistically sound framework. This approach enables researchers to clearly identify the strengths and weaknesses of analytical methods without increasing experimental complexity or cost. Its advantages in standardization， interpretability， and decision-making support its broader adoption for validating non-standard methods in research and applied laboratories.

为了获得准确可靠的实验测定数据，检测方法应用前对其进行方法确认是必需的过程。方法确认是指实验室通过试验，提供客观有效证据证明特定检测方法满足预期的用途^［1］^。方法确认的目的是确保检测方法能够获得准确可靠的结果。尽管方法确认往往耗时耗力，但若执行不充分，不仅可能造成资源浪费（包括人力、资金和材料），更可能导致暴露评估结论偏误^［2］^。在化学分析测试领域，部分受法规监管的检测实验室通过遵循如药物非临床研究质量管理规范（Good Laboratory Practice， GLP）、药物临床试验质量管理规范（Good Clinical Practice， GCP）和实验室认可准则等体系，满足外部监管机构对检测结果可靠性和可追溯性的要求^［3］^。美国食品药品监督管理局和欧洲药品管理局定期更新方法确认导则指导被监管对象获取可靠数据。然而，这些规范对于大多数科研实验室并不具有强制性约束力，因此科研人员如何选择合适的检测方法评估程序，确保所得数据的科学性与可用性，尤为关键。同时，该类用于科研目的的检测方法最终目标是在临床上开展技术应用，虽然研究工作常在科研实验室环境下进行，但其所采用的方法评估框架与质量控制理念可迁移至临床实验室实践，从而有助于提升临床检测方法的质量管理水平。目前，化学分析方法准确度评估的方法主要有3大类：一是依据已有导则或指南对方法特性参数进行确认^［4］^，二是基于测量不确定度评估指南（Guide for the Expression of the Uncertainty of Measurement， GUM）的框架^［5］^，三是基于实验验证数据的法国药学科学与技术学会（Société Française des Sciences et Techniques Pharmaceutiques， SFSTP）方法^［4］^。其中，SFSTP方法因操作简便、评估结果直观易于解释，逐渐受到关注^［6，7］^。因此，本实验室以目前日益受到关注的新污染物酚类化合物为例^［8-10］^，开展了化学分析方法准确度的评估工作。

新污染物酚类化合物作为一类典型的环境内分泌干扰物（endocrine disrupting chemicals， EDCs），对人群健康的危害越来越受到关注^［11-13］^。尿液中酚类化合物（尿酚）检测是评价人群暴露的有效手段。本实验室建立了高效液相色谱-串联质谱（high performance liquid chromatography-tandem mass spectrometry， HPLC-MS/MS）同时测定人尿液中20种环境酚类化合物的分析方法^［14］^。通过方法确认实验方案的设计，在实验过程中获得验证数据，并运用SFSTP方法绘制准确度曲线（accuracy profile， AP），进而评估所建HPLC-MS/MS分析方法的准确性与可靠性。

## 1 准确度评估的基本原理

### 1.1 测量误差相关基本概念

在利用分析方法对样本进行检测的过程中不可避免地会引入测量误差。测量误差是测量结果与被测量真值之差。由于真值不能确定，实际上用约定真值来估计并计算误差。误差按其性质，可以分为系统误差和随机误差。系统误差是指在重复性条件下对同一被测量进行无限多次测量所得结果的平均值与被测量的真值之差。随机误差是指测量结果与在重复性条件下对同一被测量进行无限多次测量所得结果的平均值之差。误差是随机误差和系统误差的代数和，理论上的测量结果是真值、随机误差、系统误差之和。测量准确度表示测量结果与被测量的真值之间的一致程度，是基于测量误差对测量结果影响的一种定性描述，是对分析方法符合预期判定标准与否的判断。测量不确定度表征合理地赋予被测量之值的分散性，与测量结果相联系的参数是在考虑了测量误差和一定置信水准等因素基础上评定出的一个区间。测量误差、准确度、不确定度之间的关系见图1。

**图1:**
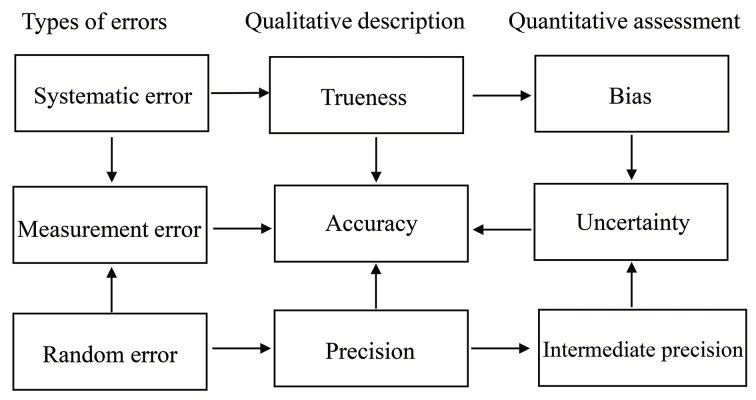
测量误差、准确度、不确定度之间的关系

### 1.2 利用SFSTP方法进行分析方法准确度评估的步骤^［4，15］^

#### 1.2.1 方法确认实验方案的一般设计

经前处理和仪器条件优化后，初步建立了满足预期目的的化学分析方法，并获得了满意的标准曲线。设置低、中、高3个方法确认标准样品浓度（*j*），安排不同批次（*i*）测定，每批中每个浓度重复测定至少3次（*k*），采集仪器响应值并根据标准曲线方程计算出相应的浓度（测定结果），用于方法准确度评估。

#### 1.2.2 精密度（precision）估计

每个浓度水平的单次测定结果根据方法误差来源可由式（1）表达：


$$X_{ijk}=\mu_{j}+\alpha_{ij}+\varepsilon_{ijk}$$ (1)

其中，*X_ijk_* 为第*i*批次、第*j*个浓度水平、第*k*次的测定结果；*μ_j_* 为第*j*浓度水平测定结果的平均值； α *_ij_* 表示在*j*浓度水平下，第*i*批次的平均值与总体均值*μ_j_* 的偏差，下文用 $\sigma_{B,j}^{2}$ 表示，其中B表示Between，即批间；*ε_ijk_* 是批内测定结果的批内相对标准偏差，下文用 $\sigma_{W,j}^{2}$ 表示，其中W表示Within，即批内。

假定测量误差在批次间是独立的。 $\sigma_{B,j}^{2}$ 为批间方差，表示批间精密度； $\sigma_{W,j}^{2}$ 为批内方差，表示批内精密度，可用均方分析法进行估计，公式见式（2）、式（3）：


$$MSM_{j}=\frac{1}{p-1}\sum_{i=1}^{p}n_{ij}({\bar{x}}_{ij,calc}-{\bar{x}}_{j,calc}{)}^{2}$$ (2)

$$MSE_{j}=\frac{1}{\sum_{i=1}^{p}n_{ij}-p}\sum_{i=1}^{p}\sum_{k=1}^{n_{ij}}(x_{ijk,calc}-{\bar{x}}_{ij,calc}{)}^{2}$$ (3)

其中，MSM为批间相对标准偏差（RSD），反映不同批次之间测量结果的RSD；MSE为批内RSD，反映同一批次内重复测量之间的RSD；*p*为试验批次数；*n*为各浓度水平批内重复测量次数；calc表示经标准曲线方程计算获得的测定结果。

*j*浓度水平的方差可以按照式（4）、式（5）估计，假如 $MSE_{j}<MSM_{j}$ ，则：


$${\hat{\sigma }}_{W,j}^{2}=MSE_{j}$$ (4)

$${\hat{\sigma }}_{B,j}^{2}=\frac{MSM_{j}-MSE_{j}}{n}$$ (5)

否则得到式（6）、式（7）：


$${\hat{\sigma }}_{W,j}^{2}=\frac{1}{pn-1}\sum_{i=1}^{p}\sum_{j=1}^{k}(x_{ijk,calc}-{\bar{x}}_{ij,calc}{)}^{2}$$ (6)

$${\hat{\sigma }}_{B,j}^{2}=0$$ (7)

批内方差也可表示为重复性方差（repeatability variance， ${\hat{\sigma }}_{Re,j}^{2}$ ），见式（8）；而将批内方差与批间方差相加，即可得到中间精密度方差（intermediate precision variance， ${\hat{\sigma }}_{Ip,j}^{2}$ ），表示总的精密度水平，见式（9）。


$${\hat{\sigma }}_{Re,j}^{2}={\hat{\sigma }}_{W,j}^{2}$$ (8)

$${\hat{\sigma }}_{Ip,j}^{2}={\hat{\sigma }}_{W,j}^{2}+{\hat{\sigma }}_{B,j}^{2}$$ (9)

#### 1.2.3 正确度（trueness）估计

分析方法的正确度表示某个浓度水平测定结果的平均值与理论真值（加标浓度值， ${\bar{x}}_{j}$ ）之间的一致程度，也称偏倚（bias），见式（10）。偏倚可以用偏差（式（11））或相对误差（式（12）表示。


$${\hat{\mu }}_{j}=\frac{1}{\sum_{i=1}^{p}n_{ij}}\sum_{i=1}^{p}\sum_{k=1}^{n_{ij}}x_{ijk,calc}$$ (10)

$$bias_{j}={\hat{\mu }}_{j}-{\bar{x}}_{j}$$ (11)

$$bias_{j}(\%)=100\times \frac{{\hat{\mu }}_{j}-{\bar{x}}_{j}}{{\bar{x}}_{j}}$$ (12)

#### 1.2.4 准确度（accuracy）估计

##### 1.2.4.1 总误差（total error）估计

每个测定结果与真值 μ 、偏倚、方法精密度之间的关系表达式见式（13）~式（15）。


$$x_{i}=\mu +{\left|bias\right|}_{procedure}+intermediate\;precision_{procedure}$$ (13)

$$x_{i}-\mu ={\left|bias\right|}_{procedure}+intermediate\;precision_{procedure}$$ (14)

$$x_{i}-\mu =total\;error_{procedure}$$ (15)

总误差（测量误差）可用于评估分析方法获得准确可靠结果的能力，它与同一浓度水平下单个测定结果的最大误差密切相关。总误差综合反映了方法准确度，可以用作方法准确度的简单初步评估。

##### 1.2.4.2 *β*-容许区间的计算^［5］^

对于分析方法准确度的评估，不仅要关注由验证数据计算的总误差（测量误差）大小，而更为关键的是要考虑将来日常分析中所获得测定结果的准确可靠性。可以用*μ_j_* 、 $\sigma_{B,j}^{2}$ 、 $\sigma_{W,j}^{2}$ 估计每个浓度水平的测定结果落在可接受限［-*λ*， +*λ*］内的预期比例，并根据Mee氏*β*‐容许区间计算方法估计准确度的范围，见式（16）~式（17）。


$$E_{\hat{\mu },\hat{\sigma }}\left\{P\left[\left|X-\mu_{T}\right|<\lambda \right]\left.{\hat{\mu }}_{M},{\hat{\sigma }}_{M}\right\}\right.\geq \beta$$ (16)

$$E_{{\hat{\mu }}_{M},{\hat{\sigma }}_{M}}\left\{P_{X}\right.\left.\left[{\hat{\mu }}_{M}-k{\hat{\sigma }}_{M}<X<{\hat{\mu }}_{M}+k{\hat{\sigma }}_{M}\left|{\hat{\mu }}_{M},{\hat{\sigma }}_{M}\right|\right]\right\}=\beta$$ (17)

其中， $E_{\hat{\mu },\hat{\sigma }}$ 表示对估计的均值 $\hat{\mu }$ 和标准差 $\hat{\sigma }$ 的期望，反映方法的不确定性；*X*表示单次测量结果； $\mu_{T}$ 表示真值； ${\hat{\mu }}_{M}$ 表示方法均值的估计值； ${\hat{\sigma }}_{M}$ 表示方法标准差的估计值；*β*表示容许的置信水平。 $E_{{\hat{\mu }}_{M},{\hat{\sigma }}_{M}}$ 表示对估计的均值 ${\hat{\mu }}_{M}$ 和标准差 ${\hat{\sigma }}_{M}$ 的联合分布求期望； k ，表示区间半宽的倍数系数。

*β*‐容许区间绝对值计算见式（18）~式（21）：


$$\left[{\hat{\mu }}_{j}-Q_{t}\left(v;\frac{1+\beta }{2}\right)\sqrt[{}]{1+\frac{1}{pnB_{j}^{2}}}{\hat{\sigma }}_{Ip,j};\;{\hat{\mu }}_{j}+\;Q_{t}\left(v;\frac{1+\beta }{2}\right)\sqrt[{}]{1+\frac{1}{pnB_{j}^{2}}}{\hat{\sigma }}_{Ip,j}\right]$$ (18)

其中：


$$R_{j}=\frac{{\hat{\sigma }}_{B,j}^{2}}{{\hat{\sigma }}_{W,j}^{2}}$$ (19)

$$B_{j}=\sqrt[{}]{\frac{R_{j}+1}{nR_{j}+1}}$$ (20)

$$v=\frac{{\left(R+1\right)}^{2}}{{\left(R+\left(1/n\right)\right)}^{2}/(p-1)+(1-(1/n))/pn}$$ (21)

$Q_{t}\left(v;\frac{1+\beta }{2}\right)$ 的*β*‐容许区间相对值计算见式（22）：


$$\left[bias_{j}\left(\%\right)-Q_{t}\left(v;\frac{1+\beta }{2}\right)\sqrt[{}]{1+\frac{1}{pnB_{j}^{2}}}CV_{Ip,j};bias_{j}\left(\%\right)+Q_{t}\left(v;\frac{1+\beta }{2}\right)\sqrt[{}]{1+\frac{1}{pnB_{j}^{2}}}CV_{Ip,j}\right]$$ (22)

其中， $R_{j}$ 表示批间RSD与批内RSD的方差比； $B_{j}$ 表示权重系数，反映了重复性和中间精密度之间的平衡； v 表示自由度； ${\hat{\mu }}_{j}$ 为第*j*个浓度水平下的观测均值； $\;{\hat{\sigma }}_{Ip,j}$ 为第*j*个浓度水平下的中间精密度标准差估计值；*p*为批次数；*n*为每批的测量重复次数； $Q_{t}\left(v;\frac{1+\beta }{2}\right)$ 为自由度*υ*下的Student’s *t*分布中的*β*分位数值。 $CV_{Ip,j}$ 为第*j*个水平的中间精密度RSD。

*β*‐容许区间包含了反映分析方法准确度的偏倚和精密度两个主要指标，同时也考虑了根据现有数据预测将来的测定结果落入（或超出）可接受限的机会（或风险），从而客观反映分析方法的准确度。

##### 1.2.4.3 准确度曲线绘制及评估

通过式（22）计算出各浓度水平的*β*‐容许区间的上、下限，并绘制在曲线上，观察落入（或超出）可接受限［-*λ*， +*λ*］的情况，进而对方法准确度进行综合评估。

### 1.3 相对总误差（relative total error， RTE）估计

相对总误差是系统误差和随机误差共同作用的结果，可以按式（23）估计。

RTE*=*∣RE∣+*z⋅*RSD (23)

其中RE为相对误差（%），RSD 为测定结果的RSD（%），*z*为与置信水平对应的正态分布双侧分位数。

### 1.4 最大观察误差（maximum observed error， MOE）估计

最大观察误差指在方法验证实验中获得的最大单次相对误差绝对值，可以按式（24）估计。

MOE=max *_i_* ∣RE *_i_* ∣ (24)

其中RE为第*i*次测定结果相对于参考值的相对误差（%）。MOE代表了在既定实验条件下方法表现出的极端误差。

### 1.5 合成后的中间精密度（ $\sigma_{Ip}$ ）估计

合成后的中间精密度是指考虑不同批次、不同人员、不同时间等条件下测量误差的总体标准差，是方法内部变异性的综合反映，见式（25）。


$$\sigma_{Ip}=\sqrt[{}]{{\sigma_{B}}^{2}+{\sigma_{W}}^{2}}$$ (25)

其中， $\sigma_{B}$ 为批间标准差； $\sigma_{W}$ 为批内标准差。

## 2 实验部分

### 2.1 仪器与试剂

1290 Infinity Series高效液相色谱仪和6490A三重四极杆质谱仪（Agilent公司，美国）。20种环境酚类标准品包括：对羟基苯甲酸甲酯（纯度为99.8%，MeP）、对羟基苯甲酸乙酯（纯度为99.9%，EtP）、对羟基苯甲酸丙酯（纯度为98.7%，PrP）、对羟基苯甲酸丁酯（纯度为99.9%，BuP）、对羟基苯甲酸庚酯（纯度为99.0%，HeP）、双酚A（纯度为99.8%，BPA）、双酚B（纯度为99.5%，BPB），双酚F（纯度为99.8%，BPF）、双酚S（纯度为99.3%，BPS）和三氯生（纯度为99.0%，TCS），购自德国Dr. Ehrenstorfer公司；对羟基苯甲酸苄酯（纯度为98%，BzP）、双酚AF（纯度为98%，BPAF）、双酚P（纯度为98%，BPP）、双酚Z（纯度为98%，BPZ）、二苯酮-1（纯度为95%，BP-1）、二苯酮-2（纯度为98%，BP-2）、二苯酮-8（纯度为98%，BP-8）和4-羟基二苯甲酮（纯度为98%，4-HBP），购自加拿大TRC公司；双酚AP（纯度为98%，BPAP）和二苯酮-3（纯度为98%，BP-3）购自美国AccuStandard公司。内标包括：MeP-^13^C_6_（加拿大，TRC公司，纯度为98%）、BPA-d_16_（加拿大，CDN公司，纯度为98%）、BP-3-d_5_（上海甄准生物科技有限公司，纯度为98%）、TCS-d_3_（德国，Dr. Ehrenstorfer公司，纯度为98%）。HPLC级甲醇和乙酸乙酯购自德国Merck公司。

### 2.2 仪器分析方法

液相色谱条件 Poroshell 120 EC-C18色谱柱（100 mm×3.0 mm，2.7 μm），柱温40 ℃，流速为0.5 mL/min，进样体积为5 μL。流动相由纯水和甲醇组成，梯度洗脱程序如下：初始为25%甲醇，在5.0 min时升至95%甲醇 并保持至11.0 min，随后于11.1 min恢复至25%甲醇，并维持至13.0 min结束。

质谱条件 采用负离子电喷雾离子源（electrospray ionization，ESI）结合多反应监测模式（multiple reaction monitoring， MRM）进行检测。离子源温度设为250 ℃，毛细管电压为3 000 V，雾化气压力为29 MPa。具体质谱参数见表1。

**表1:** 目标物的母离子、子离子及优化后的碰撞能

Compound	Precursor ion （*m/z*）	Quantification		Qualification	
		Q3 （*m/z*）	CE/eV	Q3 （*m/z*）	CE/eV
MeP	151.0	92.0	22	136.0	14
EtP	165.0	92.0	15	137.0	12
PrP	178.9	91.8	21	137.0	19
BuP	192.9	91.8	19	136.0	15
BzP	227.0	92.0	18	136.1	10
HeP	235.0	92.0	31	135.9	25
BPA	227.0	133.0	27	210.9	20
BPAF	334.7	265.0	20	/	/
BPAP	289.0	273.9	20	/	/
BPB	241.2	211.7	21	223.0	21
BPF	198.8	93.0	21	104.9	21
BPP	345.0	328.9	37	315.0	41
BPS	248.9	108.0	27	91.7	33
BPZ	266.9	173.1	29	223.1	33
BP-1	213.0	134.9	21	91.0	22
BP-2	245.0	135.0	15	109.1	18
BP-3	227.1	211.1	20	167.0	20
BP-8	243.1	123.1	15	93.1	19
4-HBP	197.1	92.1	33	169.2	31
TCS	287.0	35.1	9	/	/
MeP-^13^C_6_	156.8	98.1	20	142.0	16
BPA-d_16_	241.1	142.1	30	222.0	26
BP-3-d_5_	232.0	214.7	20	/	/
TCS-d_3_	291.9	35.1	9	/	/

CE： collision energy. MeP： methyl 4-hydroxybenzoate； EtP： ethyl 4-hydroxybenzoate； PrP： propyl 4-hydroxybenzoat； BuP： butyl 4-hydroxybenzoate； BzP： benzyl 4-hydroxybenzoate； HeP： heptyl 4-hydroxybenzoate； BPA： bisphenol A； BPAF： bisphenol AF； BPAP： bisphenol AP； BPB： bisphenol B； BPF： bisphenol F； BPP： bisphenol P； BPS： bisphenol S； BPZ： bisphenol Z； BP-1： 2，4-dihydroxy benzophenone； BP-2： 2，2′，4，4′-tetrahydroxy benzophenone； BP-3： 2-hydroxy-4-methoxybenzophenon； BP-8： 2，2′-dihydroxy-4-methoxy benzophenone-dihydroxy-4-methoxy benzophenone； 4-HBP： 4-hydroxy benzophenone； TCS： triclosan.

### 2.3 前处理方法

取200 μL尿液样本，加入一定量的内标工作液和酶工作液，涡旋混匀后，于37 ℃条件下孵育过夜。孵育完成后，向样品中加入500 μL乙酸乙酯，涡旋混匀，并于4 ℃、15 000 r/min条件下离心10 min。取上层有机相转移至EP管。对残余尿液重复上述萃取步骤，再次取上层有机相合并，作为最终萃取液。将合并后的有机相于40 ℃下使用离心浓缩仪浓缩至完全干燥。随后加入200 μL的80%甲醇水溶液，涡旋混匀，以充分重溶。样品再经4 ℃、15 000 r/min离心3 min，收集上清液，转入内插管色谱瓶中，用于后续的HPLC-MS/MS分析。本研究已获得上海交通大学医学院附属新华医院伦理委员会批准（XHEC-C-2013-001）。具体实验流程参考前期发表的方法学文章（文献［^14^］）。

### 2.4 数据处理

采用Minitab17.1.0软件（Minitab公司，美国）进行数据处理和统计分析。

## 3 结果与讨论

### 3.1 工作曲线的线性关系

以空白尿液为基质，配制7个不同浓度的标准溶液系列，构建工作曲线并计算线性方程。各组分在0.5~100 ng/mL的范围内获得了良好的线性关系，相关系数为0.991 0~0.999 7（见表2），可以用于后续验证数据的采集。

**表2:** 20种尿酚的线性范围、回归方程及相关系数

Compound	Linear range/（ng/mL）	Regression equation	*r* ^2^
MeP	0.5-100	*Y*=-0.00001+0.07106*X*	0.9992
EtP	0.5-100	*Y*=0.02151*X*	0.9991
PrP	0.5-100	*Y*=-0.00002+0.02935*X*	0.9975
BuP	0.5-100	*Y*=-0.00003+0.03704*X*	0.9924
BzP	0.5-100	*Y*=0.23230*X*	0.9919
HeP	0.5-100	*Y*=0.1130+0.3901*X*	0.9911
BPA	0.5-100	*Y*=0.000003+0.009441*X*	0.9987
BPAF	0.5-100	*Y*=0.9156*X*	0.9995
BPAP	0.5-100	*Y*=0.1639*X*	0.9989
BPB	0.5-100	*Y*=0.01625*X*	0.9989
BPF	1.0-100	*Y*=0.000038+0.000256*X*	0.9988
BPP	0.5-100	*Y*=-0.00002+0.01452*X*	0.9943
BPS	0.5-100	*Y*=0.4319*X*	0.9985
BPZ	0.5-100	*Y*=0.00004+0.03775*X*	0.9997
BP-1	0.5-100	*Y*=0.1073+0.3812*X*	0.9994
BP-2	0.5-100	*Y*=0.2479+0.4954*X*	0.9991
BP-3	1.0-100	*Y*=-0.000105+0.003505*X*	0.9985
BP-8	0.5-100	*Y*=-0.00004+0.06278*X*	0.9910
4-HBP	0.5-100	*Y*=0.04842+0.07528*X*	0.9994
TCS	0.5-100	*Y*=-0.00005+0.02946*X*	0.9990

*Y：* peak area ratios of phenols to internal standards；*X：* mass concentration ratios of phenols to internal standard.

### 3.2 精密度评估

根据精密度的计算公式（2）~（9），在低、中、高3个浓度水平下，20种尿酚检测方法的批内和批间RSD分别为2.8%~10.7%和3.3%~14.4%，合成后的中间精密度RSD为4.5%~16.6%（见表3）。可以看出，RSD均不超过20%，说明该方法的重复性结果比较满意。

**表3:** 20种尿酚分析方法的精密度

Compound	Intra-batch RSDs/%			Inter-batch RSDs/%			Intermediate RSDs/%		
	1^*^	10^*^	50^*^	1^*^	10^*^	50^*^	1^*^	10^*^	50^*^
MeP	3.7	3.6	3.7	8.9	6.3	3.3	9.7	7.3	4.9
EtP	4.6	3.8	3.5	8.5	7.2	3.5	9.7	8.2	4.9
PrP	6.9	3.8	3.8	7.3	6.8	7.3	10.1	7.8	8.2
BuP	4.4	3.7	4.0	3.9	6.4	5.8	5.6	7.4	7.0
BzP	4.1	3.7	4.0	3.9	6.4	5.8	5.6	7.4	7.0
HeP	5.2	2.8	4.5	7.0	3.5	5.8	8.7	4.5	7.4
BPA	8.3	5.8	5.4	14.4	7.4	5.0	16.6	9.4	7.4
BPAF	4.8	4.3	5.4	6.3	7.0	5.6	7.9	8.2	7.8
BPAP	6.4	6.9	4.4	5.6	8.2	4.9	8.5	10.7	6.6
BPB	3.5	5.6	6.2	7.3	6.8	5.1	8.1	8.8	8.1
BPF	3.4	8.9	7.5	8.7	10.8	9.0	9.3	14.1	11.7
BPP	6.2	5.3	5.5	8.7	7.1	7.3	10.7	8.9	9.1
BPS	3.3	4.7	4.1	5.7	8.0	8.4	6.5	9.3	9.4
BPZ	7.9	6.0	5.1	8.7	7.9	7.6	11.8	9.9	9.1
BP-1	6.4	5.7	7.0	8.1	9.1	9.7	10.3	10.8	11.9
BP-2	4.4	5.5	7.9	6.2	8.4	7.3	7.6	10.1	10.7
BP-3	7.5	6.5	7.1	8.9	7.8	11.8	11.7	10.1	13.7
BP-8	5.1	5.5	10.7	5.8	7.3	9.5	7.7	9.1	14.4
4-HBP	3.6	4.1	5.9	6.3	7.9	8.8	7.2	8.9	10.6
TCS	4.8	3.4	4.0	6.7	5.4	3.6	9.7	7.3	4.9

* ng/mL.

### 3.3 正确度估计

经式（10）~（12）计算，在低、中、高3个浓度水平下，20种尿酚检测方法的相对误差为-25.3%~13.0%（见表4）。值得注意的是，BzP在低水平（1 ng/mL）的相对误差为-25.3%，显著偏离其他化合物，提示该化合物在低水平下可能存在系统性低估的趋势。可能原因包括基质效应增强负离子抑制，或目标化合物的低回收率问题，这在以LC-MS/MS为检测方法的环境暴露生物监测研究中已有类似报道^［16，17］^。Matuszewski等^［18］^的研究指出，在负离子电喷雾模式下，尿液等复杂基质更容易引发离子抑制，尤其在多组分同时检测时更为显著，进而导致信号强度降低，造成目标化合物浓度被系统性低估。另一方面，BzP在本方法中未使用同位素标记的专属内标，仅依赖类BPA内标进行校正，可能无法充分抵消基质干扰和信号波动，从而进一步加剧了低浓度测定值的系统性低估。因此，未来在方法优化中可考虑进一步增强BzP的萃取效率、更换对应同位素内标、提高校正能力进而加以修正。

**表4:** 20种尿酚测定的方法相对误差、精密度、相对总误差及最大观察误差

Compound	Relative error/%			Intermediate precision （RSDs/%）			Relative total error/%			Maximum observation error/%		
	1 ng/mL	10 ng/mL	50 ng/mL	1 ng/mL	10 ng/mL	50 ng/mL	1 ng/mL	10 ng/mL	50 ng/mL	1 ng/mL	10 ng/mL	50 ng/mL
MeP	-10.7	3.6	2.5	9.7	7.3	4.9	20.3	10.8	7.5	26.8	15.5	13.8
EtP	-2.9	-3.1	-0.7	9.7	8.2	4.9	12.6	11.3	5.6	18.7	16.2	10.3
PrP	-0.7	3.7	0.7	10.1	7.8	8.2	10.7	11.5	8.9	23.7	16.7	18.3
BuP	-12.3	9.2	-1.8	5.6	7.4	7.0	17.9	16.6	8.9	26.2	24.1	12.4
BzP	-25.3	1.0	-2.5	5.6	7.4	7.0	30.9	8.4	9.5	37.5	16.7	14.8
HeP	8.0	-4.1	-2.5	8.7	4.5	7.4	16.7	8.6	9.9	24.9	11.7	17.3
BPA	13.0	1.3	2.9	16.6	9.4	7.4	29.6	10.7	10.3	53.0	24.3	14.9
BPAF	-0.8	2.0	0.8	7.9	8.2	7.8	8.7	10.2	8.6	17.5	17.8	17.4
BPAP	-0.7	7.0	1.8	8.5	10.7	6.6	9.2	17.7	8.4	18.5	30.8	16.2
BPB	-5.9	0.8	0.1	8.1	8.8	8.1	14.0	9.6	8.1	20.3	21.9	18.7
BPF	-18.3	-5.4	-2.1	9.3	14.1	11.7	27.6	19.4	13.8	34.2	29.4	27.7
BPP	10.9	7.0	1.3	10.7	8.9	9.1	21.6	15.8	10.4	33.9	24.4	23.0
BPS	-24.3	-15.9	0.6	6.5	9.3	9.4	30.8	25.2	10.0	38.5	27.5	21.7
BPZ	3.9	7.1	3.1	11.8	9.9	9.1	15.7	17.0	12.2	29.2	29.7	17.8
BP-1	5.0	4.4	-2.2	10.3	10.8	11.9	15.3	15.2	14.2	36.6	25.5	20.9
BP-2	-9.9	-2.2	0.1	7.6	10.1	10.7	17.5	12.3	10.9	25.8	18.8	19.7
BP-3	3.6	6.3	4.4	11.7	10.1	13.7	15.3	16.4	18.2	32.6	40.0	27.1
BP-8	-22.9	-6.5	-0.3	7.7	9.1	14.4	30.7	15.7	14.7	34.9	21.0	30.0
4-HBP	-14.7	-6.9	5.1	7.2	8.9	10.6	21.9	15.8	15.8	29.5	25.3	23.5
TCS	-10.7	3.6	2.5	9.7	7.3	4.9	20.3	10.8	7.5	29.4	12.9	13.0

### 3.4 准确度评估

#### 3.4.1 总误差估计

经式（13）~（15）计算，在低、中、高3个浓度水平，20种尿酚检测方法的相对总误差为5.6%~30.9%，最大观察误差为10.3%~53.0%（见表4）。该方法在不同浓度水平的相对总误差与同一浓度水平下单个测定结果的最大误差密切相关（*R*=0.839，*p*<0.000 1）（见图2）。20种尿酚的相对总误差中，95%的结果落在预定接受限（70%~130%）内（见表4），初步判断方法的总体准确度尚可。此外，表4显示BPA在1 ng/mL时的相对总误差为29.6%，接近30%的可接受限，最大观察误差更达到53.0%。虽然该结果仍在可接受范围内，但其临界值特征表明该方法在低浓度水平下对BPA的检测准确性具有一定不确定性风险。

**图2:**
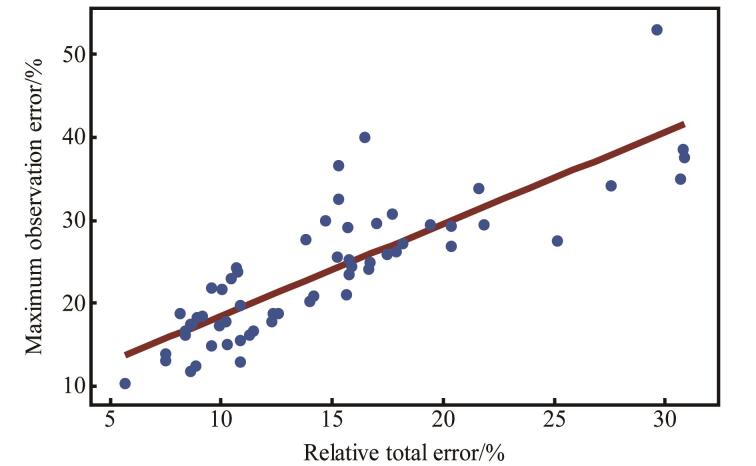
不同浓度水平的相对总误差与最大观察误差的相关性

#### 3.4.2 *β*-容许区间的计算

将分析方法的准确度可接受限设定为30%，*β*置信水平设为95%，通过验证实验数据并结合Mee氏*β*‐容许区间计算方法及式（22），该检测方法在不同浓度水平的*β*‐容许区间为-37.99%~45.19%（见表5），13种酚类在各浓度水平均满足可接受范围设定值，其余7种在低或中浓度水平超出设定值。

**表5:** 20种尿酚测定方法的 ***β*** ‐容许区间

Compound	Average mass concentration of measurement results/（ng/mL）			*β*-Tolerance interval/（ng/mL）			*β-*Tolerance interval/%			
	1ng/mL	10ng/mL	50ng/mL	1 ng/mL	10 ng/mL	50 ng/mL	1 ng/mL	10 ng/mL	50 ng/mL	
MeP	0.89	10.36	51.27	［0.70， 1.08］	［8.95， 11.77］	［46.81， 55.73］	［-29.76， 8.43］	［-10.53， 17.66］	［-6.39， 11.45］	
EtP	0.97	9.69	49.66	［0.71， 1.08］	［8.77， 11.94］	［46.79， 55.74］	［-21.62， 15.78］	［-18.93， 12.71］	［-9.63， 8.27］	
PrP	0.99	10.37	50.34	［0.81， 1.18］	［8.86， 11.87］	［42.40， 58.28］	［-19.15， 17.85］	［-11.41， 18.74］	［-15.21， 16.56］	
BuP	0.88	10.92	49.09	［0.78， 0.98］	［9.49， 12.35］	［42.45， 55.73］	［-22.50， -2.01］	［-5.09， 22.35］	［-15.10， 11.46］	
BzP	0.75	10.10	48.75	［0.65， 0.85］	［8.66， 11.53］	［42.11， 55.39］	［-35.49， -15.00］	［-13.37， 15.27］	［-15.78， 10.77］	
HeP	1.08	9.59	48.75	［0.92， 1.24］	［8.75， 10.43］	［41.87， 55.62］	［-8.36， 24.36］	［-12.53， 4.34］	［-16.26， 11.24］	
BPA	1.13	10.13	51.43	［0.81， 1.45］	［8.37， 11.88］	［44.72， 58.15］	［-19.20， 45.19］	［-16.33， 18.84］	［-10.56， 16.29］	
BPAF	0.99	10.20	50.41	［0.84， 1.14］	［8.63， 11.77］	［43.27， 57.54］	［-15.54， 14.03］	［-13.70， 17.68］	［-13.46， 15.09］	
BPAP	0.99	10.70	50.89	［0.84， 1.15］	［8.71， 12.69］	［44.81， 56.96］	［-15.92， 14.54］	［-12.89， 26.87］	［-10.37， 13.92］	
BPB	0.94	10.08	50.02	［0.78， 1.10］	［8.45， 11.71］	［42.78， 57.27］	［-21.86， 10.09］	［-15.50， 17.06］	［-14.43， 14.53］	
BPF	0.82	9.46	48.97	［0.63， 1.00］	［6.86， 12.07］	［38.10， 59.83］	［-36.63， 0.14］	［-31.43， 20.69］	［-23.79， 19.66］	
BPP	1.11	10.70	50.65	［0.91， 1.31］	［9.02， 12.37］	［42.05， 59.24］	［-9.26， 31.08］	［-9.77， 23.69］	［-15.89， 18.48］	
BPS	0.76	8.41	50.31	［0.63， 0.88］	［6.62， 10.20］	［41.07， 59.56］	［-36.59， -11.63］	［-33.83， 2.05］	［-17.85， 19.11］	
BPZ	1.04	10.71	51.55	［0.82， 1.26］	［8.84， 12.58］	［42.85， 60.26］	［-17.83， 25.57］	［-11.63， 25.77］	［-14.30， 20.52］	
BP-1	1.05	10.44	48.88	［0.85， 1.25］	［8.29， 12.59］	［37.88， 59.89］	［-15.26， 25.16］	［-17.09， 25.92］	［-24.25， 19.79］	
BP-2	0.90	9.78	50.07	［0.75， 1.05］	［7.77， 11.78］	［40.57， 59.56］	［-24.91， 5.12］	［-22.30， 17.83］	［-18.85， 19.11］	
BP-3	1.04	10.63	52.22	［0.81， 1.26］	［8.67， 12.59］	［39.10， 65.33］	［-19.10， 26.29］	［-13.34， 25.93］	［-21.79， 30.66］	
BP-8	0.77	9.35	49.83	［0.62， 0.92］	［7.55， 11.15］	［36.86， 62.80］	［-37.99， -7.88］	［-24.51， 11.48］	［-26.29， 25.61］	
4-HBP	0.85	9.31	52.56	［0.71， 1.00］	［7.52， 11.10］	［42.43， 62.70］	［-29.27， -0.05］	［-24.84， 11.01］	［-15.15， 25.40］	
TCS	0.85	10.24	50.90	［0.69， 1.01］	［8.97， 11.50］	［46.04， 55.77］	［-31.10， 1.39］	［-10.33， 15.04］	［-7.92， 11.54］	

#### 3.4.3 准确度曲线绘制及评估

准确度曲线（图3）显示，BPAF、BPB等14种尿酚在不同浓度水平下的*β*-容许区间均落在±30%以内，95%置信水平下准确度可接受，分析方法准确可靠。4种尿酚在低浓度水平的*β*-容许区间超出了接受限，当质量浓度分别提升至3.18 ng/mL（BzP）、6.01 ng/mL（BPA）、6.43 ng/mL（BP-8）、1.43 ng/mL（TCS）时，方法准确度在可接受限30%内，测定结果准确。BPF和BPS 2种尿酚在低、中浓度水平的*β*-容许区间均超出了接受限，当质量浓度分别提升至18.4 ng/mL、19.68 ng/mL时，测定结果方才准确。尽管准确度曲线结果显示大多数酚类化合物在各浓度水平下准确性良好，但仍有7种尿酚（BzP、BPA、BP-8、TCS、BPF、BPS、BPP）在低或中浓度水平的*β*-容许区间超出了预设可接受限30%。其中，BPF和BPS 2种尿酚在低、中浓度水平的*β*-容许区间均超出了接受限，准确度曲线分析表明其*β*-容许区间收敛至可接受限所需质量浓度分别为18.41 ng/mL和19.68 ng/mL，而此水平明显高于一般人群尿液中酚类化合物的实际暴露水平（通常低于1.2 ng/mL）^［9，19］^，提示该方法在常规人群监测中可能无法可靠定量上述两种物质。此类偏差可能源于样品前处理步骤中目标化合物的提取效率不足、基质干扰引起的离子抑制效应未能完全通过内标校正消除，或仪器响应在低浓度区域的稳定性较差^［20］^。因此，当前方法在中高浓度区间的准确性较好，适用于暴露水平相对较高的人群或特定研究场景，但在超低暴露背景下对个别化合物的定量准确性仍存局限，需结合实际研究目的权衡使用或进一步优化。

**图3:**
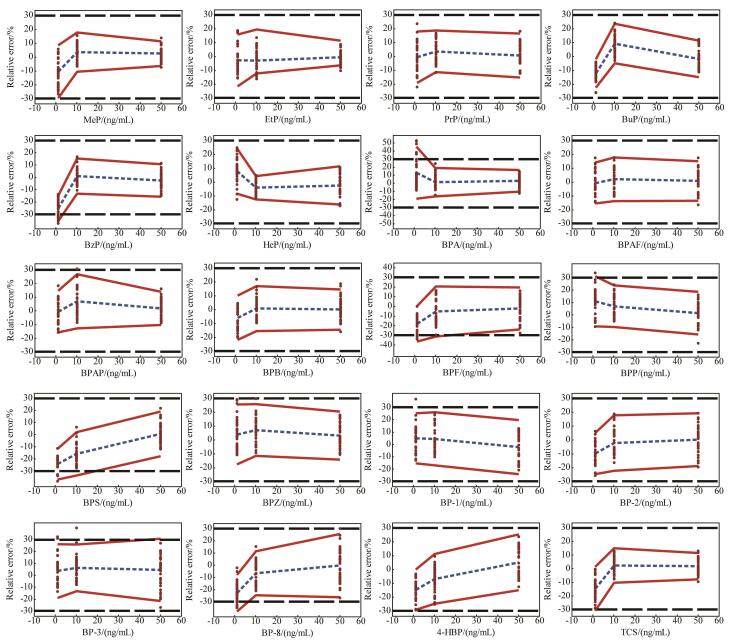
20种尿酚的准确度曲线

## 4 结论

本研究基于准确度曲线对20种尿酚的测定方法开展系统性准确度评估，结果显示所建立的HPLC-MS/MS定量分析方法在科研实验室条件下亦能实现较高的准确性与可靠性。相比传统依赖单一特性参数的评价方式，准确度曲线方法融合方法学性能评估与风险管理理念，具备评价维度全面、结果展示直观、统计标准统一等优势。同时，该策略无需增加实验成本，仅依赖一次完整的统计分析即可明确判断方法是否适用于既定目的。值得指出的是，本研究不仅验证了该方法在科研环境中的可行性和实用性，更展示了该评估框架在质量控制体系构建中的广泛适应性。其理念与实施路径对推动临床和公共卫生实验室质量管理的标准化、规范化具有借鉴意义，亦为环境暴露检测方法向临床应用延伸提供了方法学支持。
